# Convolutional Neural Networks in the Diagnosis of Cervical Myelopathy

**DOI:** 10.1055/s-0044-1779317

**Published:** 2024-12-07

**Authors:** Murat Korkmaz, Hakan Yılmaz, Merve Damla Korkmaz, Turgut Akgül

**Affiliations:** 1 Departamento de Ortopedia e Traumatologia, Istanbul Faculty of Medicine, Istanbul University, Istambul, Turquia; 2 Departamento de Engenharia Médica, Faculty of Engineering, Karabuk University, Karabuk, Turquia; 3 Departamento de Medicina Física e Reabilitação, Istanbul Kanuni Sultan Suleyman Training and Research Hospital, University of Health Sciences, Istambul, Turquia

**Keywords:** artificial neural networks, cervical vertebrae, deep learning, magnetic resonance imaging, myelopathy

## Abstract

**Objective** Artificial intelligence technologies have been used increasingly in spine surgery as a diagnostic tool. The aim of the present study was to evaluate the effectiveness of the convolutional neural networks in the diagnosis of cervical myelopathy (CM) compared with conventional cervical magnetic resonance imaging (MRI).

**Materials and Methods** This was a cross-sectional descriptive analytical study. A total of 125 participants with clinical and radiological diagnosis of CM were included in the study. Sagittal and axial MRI images in the T2 sequence of the cervical spine were used. All image parts were obtained as 8 bytes/pixel in 2 different categories, CM and normal, both in axial and sagittal views.

**Results** Triple cross validation was performed to prevent overfitting during the training process. A total of 242 sample images were used for training and testing the model created for axial views. In the axial view, the calculated values are 97.44% for sensitivity and 97.56% for specificity. A total of 249 sample images were used for training and testing the model created for sagittal views. The calculated values are 97.50% for sensitivity and 97.67% for specificity. After the training, the average accuracy value was 96.7% (±1.53) for the axial view and 97.19% (±1.2) for the sagittal view.

**Conclusion** Deep learning (DL) has shown a great improvement especially in spine surgery. We found that DL technology works with a higher accuracy than other studies in the literature for the diagnosis of CM.

## Introduction

Cervical myelopathy (CM) is a common degenerative disease of the cervical spine caused by spinal cord compression. 1 The decrease in the volume of the spinal canal is usually due to disc degeneration, osteophytes or ossified posterior longitudinal ligament results with ischemic changes. The clinical findings of the CM vary with the severity and location of the spinal cord compression. 2 Patients generally present with numbness and pain in the extremities and neurological symptoms such as loss of coordination and balance. The clinical and radiological modalities of the patient should be evaluated together in the evaluation of this disease and in determining the treatment options. 3

The imaging procedures for CM are plain radiography, magnetic resonance imaging (MRI) and computed tomography (CT). Magnetic resonance imaging in CM is more valuable in evaluating the spinal cord, disc and other soft tissues compared with other imaging methods. 4 5 As a result of advances in MRI technology such as MR spectroscopy and diffusion tensor imaging, resolution and image quality have greatly improved. In parallel with these developments, new imaging methods are being developed with many studies in the diagnosis of CM. 6

Convolutional neural networks (CNNs), a machine learning (ML) technique, are increasingly used in various industrial and research fields. 7 Deep learning (DL) is a multi-layered neural network in which feature extraction is done automatically. It extends traditional neural networks by adding more hidden layers to the network architecture between the input and output layers to model more complex and nonlinear relationships.

Machine learning technologies are also used in many health applications such as medical image analysis, biological signal analysis, prediction of medical events, computer-aided detection and diagnosis, aiding in treatment selection, and analysis of health records for recent years. 8

Machine learning technology has been used increasingly in spine surgery as well as in many medical fields. However, it is noticed that most of the studies about this subject in the literature have been conducted except of the cervical spine. 9 In the present study, we aimed to demonstrate the effectiveness of the CNN in the diagnosis of CM compared with conventional cervical MRI.

## Materials and Methods

### Participants and MRI Samples

**Fig. 1:**
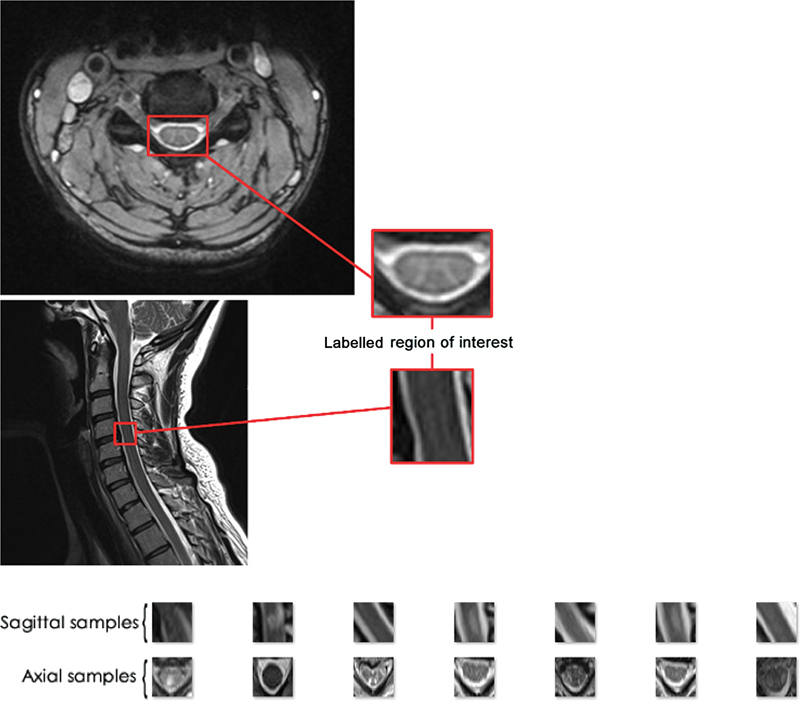
Labeled region of interest of the axial and sagittal MRI section (top) and samples of input images (bottom).

Participants aged between 18 and 75 years old diagnosed as CM were included in the study. All images were obtained with a 3.0 Tesla Siemens MR scanner (Siemens, Erlangen, Germany) with a standard cervical collar. Radiological images of the cervical spine were evaluated by three investigators experienced with a minimum experience of 5 years in spinal surgery. Axial and sagittal MRI images of the cervical spine were used. Magnetic resonance imaging scans in Digital Imaging and Communications in Medicine (DICOM) format (8 bits/pixel) have been converted to one of the raster formats (png). Thereafter, the 'region of interest' selection was made, and the regions were labeled as CM and normal, both in axial and sagittal views ( Fig. 1 ).

The inclusion criteria for the study were the participants with classical CM symptoms such as weakness and clumsiness at the upper extremities, hyperreflexia, and/or gait difficulties; radiological findings of spinal compression; scores on the modified Japanese Orthopedic Association scale (mJOA) < 18. Participants with a previous history of cervical spinal surgery and those with a systematic disease (rheumatologic or neural disease) were excluded from the study. The present study was approved by the local ethical committee (KAEK/2020.07.129) and registered at Clinicaltrials.gov (NCT04796987).

### Calculation Environment

A computer with 8 GB memory and Intel i7 processor was used to perform the calculations and train the DL model. Deep learning models were developed using the Python programming language. To assess the model performance, mean and median cross-validated accuracies, sensitivity, specificity, positive, and negative predictive value were used.

### Neural Network Design

**Fig. 2:**
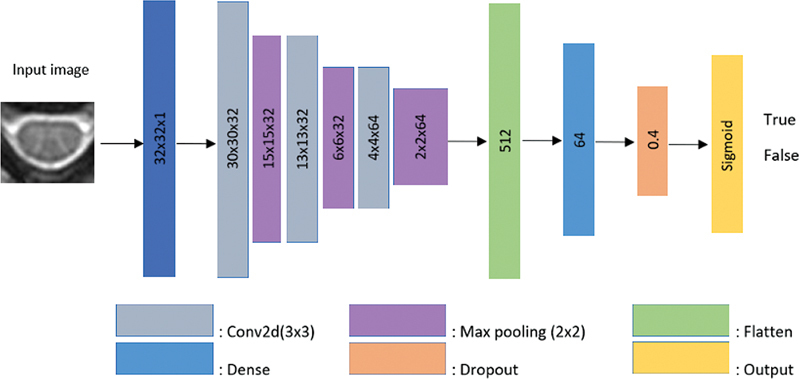
The CNN architecture used in the study.

Significant developments in ML and image processing techniques enable medical images to be evaluated more accurately using DL methods. 7 The term “deep learning” has been used for many years in the classification of medical images. Convolutional neural network, one of the popular DL methods, was used to classify images in the present study. A simple CNN architecture consists of convolutional layer (Conv), pooling layer (for example, Max-pooling), nonlinear layer (for example, ReLU, TanH) and a loss function (for example, Sigmoid, Softmax) on the last fully connected (FC). Output can be of a single class; however, it can also have multiple outputs. 10 The CNN architecture used in the study is presented in Fig. 2 .

## Results

A total of 125 participants (58 female and 67 male) with a mean age of 56.32 *±* 6.77 years old who were diagnosed as CM were included in the study. The mean mJOA scores of the participants was 12.01 ± 2.46. Demographic characteristics of the participants are shown in Table 1 . Normal and myelomalasic MRI layers of the participants were analyzed.

**Table 1:** Demographic and clinical characteristics of the participants

Variable	Participants ( *n* = 125)
Age (in years) (mean ± SD)	56.32 ± 6.77
Gender (female/male) n (%)	58 (46.4%)/67 (53.6%)
Height (cm) (mean ± SD)	164.5 ± 8.52
Weight (kg) (mean ± SD)	74.96 ± 8.48
mJOA score	12.01 ± 2.46

### Training Information

In the created network design, sample images taken in both axial and sagittal view were subjected to training. Thus, two different DL trainings were performed for both views; 67% of the images were used for training, and 33% for validation and testing. Triple cross validation was used to test the consistency of the results.

**Fig. 3:**
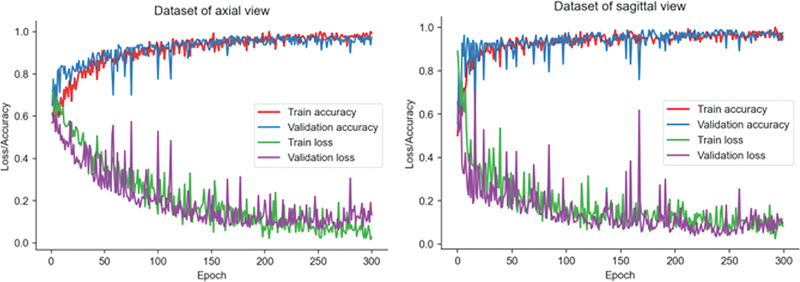
Training information of the sagittal and axial view of the MRI.

The models obtained for both views consist of 300 epochs. Triple cross validation was performed to prevent overfitting during the training process. After training, the average accuracy value was 96.7% (±1.53) for the axial view and 97.19% (±1.2) for the sagittal view. The graphs of the DL training process are shown in Fig. 3 .

### Evaluation Metrics of Training

#### Axial View

**Fig. 4:**
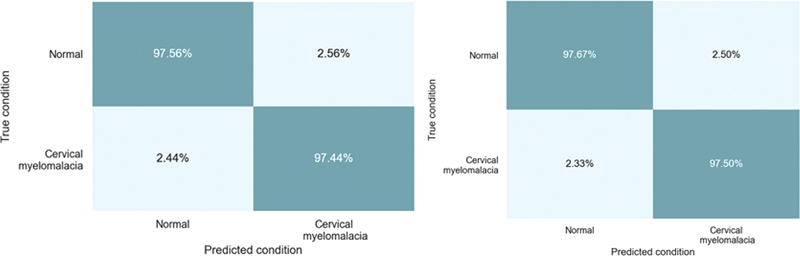
Confusion matrix of axial view (left) and sagittal view (right).

A total of 242 sample images were used for training and testing the model created for axial views. Of these images, 120 were labeled as CM, and 122 were labeled as normal. A total of 162 of these images were used for the training of the DL model, and 80 for the evaluation of the training. The true positive, true negative, false positive, and false negative values obtained from the evaluation of the training are shown in Table 2 . The confusion matrix accuracy values formed by these values are shown in Fig. 4 . After the evaluation, the overall accuracy value of the model was calculated as 97.50%.

**Table 2:** Details of the sagittal and axial images used for evaluation

Total population		Human (true condition)	
		*Condition positive*	*Condition negative*
Developed model (predicted condition) for sagittal images	*Predicted condition positive*	True positive = 39	False positive = 1 (type-I error)
	*Predicted condition negative*	False negative = 1 (type-II error)	True negative = 42
Developed model (predicted condition) for axial images	*Predicted condition positive*	True positive = 38	False positive = 1 (type-I error)
	*Predicted condition negative*	False negative = 1 (type-II error)	True negative = 40

Abbreviations: cm, centimeters; kg, kilogram; mJOA, modified Japanese Orthopedic Association scale; SD, standard deviation.

The calculated values are 97.44% for true-positive rate (TPR, recall, sensitivity), 97.56% for true-negative rate (TNR, specificity), 2.56% for false-negative rate (FNR, miss rate), and 97.44% for positive predictive value (PPV, precision). Also, the F _1_ score is calculated as 0.97, Matthew correlation coefficient (MCC) as 0.95, and Cohen kappa coherent as 0.95.

**Fig. 5:**
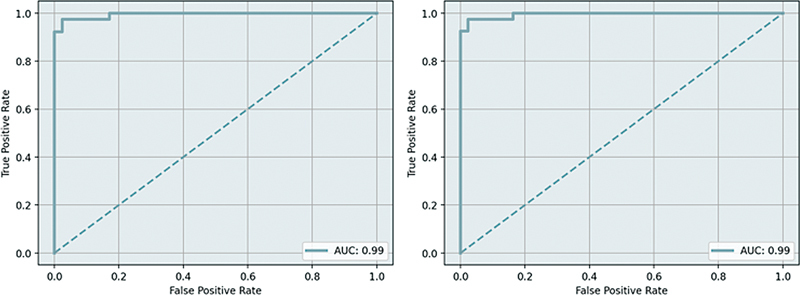
ROC curve and AUC value of the axial view (left) and sagittal view (right).

After the evaluation, the receiver operating characteristic (ROC) curve was drawn, the area under the ROC curve 11 was calculated, and shown in Fig. 5 .

#### Sagittal view

A total of 249 sample images from the MRI of the participants were selected for training and testing the model created for sagittal views. Of these images, 124 were labeled as CM, and 125 were labeled as normal. A total of 166 of these images were used for the training of the DL model, and 83 for the evaluation of the training. The true positive, true negative, false positive, and false negative values that occur after the evaluation of the training are shown in Table 2 . The confusion matrix accuracy values formed by these values are given in Fig. 4 . After the evaluation, the overall accuracy value of the model was calculated as 97.59%.

The calculated values are 97.50% for TPR (recall, sensitivity), 97.67% for TNR (specificity), 2.50% for FNR (miss rate), and 97.50% for PPV (precision). Also, the F _1_ score is calculated as 0.98, MCC as 0.95, and Cohen kappa coherent as 0.95.

After the evaluation, the ROC curve was drawn, the AUC was calculated, and shown in Fig. 5 .

## Discussion

Machine learning technologies have started to be used in many areas with the development it has shown in recent years. 12 Deep learning, a sub-branch of ML, has shown a great improvement especially in the medical field. In parallel with this development, the use of DL in spine surgery has increased significantly in recent years and has brought a new breath to the determination of spine problems and the evaluation of treatment options. 9 Machine learning is currently used more frequently in the evaluation of diagnostic imaging, such as the location of vertebra and discs and the shape of the spine, with the information obtained from radiological imaging methods such as CT and MRI. 13 However, with the progress in ML studies, its use is gradually increasing in treatment protocols and predicting possible results. 14

The diagnosis of CM is based on findings obtained in clinical examination and radiological imaging. Magnetic resonance imaging is the most effective diagnostic method in the radiological evaluation of patients with clinical myelopathic symptoms. 15 Spinal cord anatomy and medullary canal structures are better evaluated in sagittal and axial views. 16 17 Cervical myelopathy characteristically manifests itself as hypointense focal cord atrophy in the T1 sequence and hyperintense in the T2 sequence. However, it has been reported that the T2 sequence detects intramedullary changes better and is more valuable in terms of prognosis. 15 It has also been reported that between 58 and 85% of patients with clinical symptoms of CM have a hyperintense signal in the T2 sequence. 18 Evaluation of CM is primarily based on subjective judgements of the MRI findings of the spinal cord and is therefore limited by the training and expertise of the evaluator. 19 Therefore, in the present study, we tried to reveal the effectiveness of DL in the diagnosis of myelopathy to eliminate the subjection on the person at diagnosis.

In this respect, we evaluated the T2 sequence of the cervical MRI of participants with myelomalacia in the present study. In this sequence, sagittal and axial MR sections of the participants were obtained, normal and areas of myelomalacia in the intramedullary region were marked and the machine was trained. Cervical MRI of the participants were taken in a single center and with the same MRI device. However, because MRI scans may differ from patient to patient, normal images and those of the field of myelomalacia were obtained in the same section to standardize the scans.

In a pilot study, the effectiveness of ML in the diagnosis of CM was evaluated on 28 patients. In this study in which the cervical MRIs of participants with myelopathy symptoms were evaluated, the median accuracy was determined as 90%. The mean sensitivity was 90% and specificity was 85%. 20 Similarly, in another study investigating the effectiveness of ML in CM, diffusion tensor imaging was used. In this study, mean accuracy was 89.7%, sensitivity was 85%, and specificity was 92.4%. 21 In another study, the highest mean accuracy found at the end of the study was 77%, the mean sensitivity was 78%, and mean specificity was 80%. 22 In the present study, we used conventional cervical MRIs, which are used as a standard in diagnosis in a daily routine. Because the anatomy of the spinal cord is best evaluated in the axial and sagittal planes, we evaluated these two planes separately. At the end of the study, the median accuracy in the axial plane was found to be 97.50%. Average sensitivity was 97.44% and specificity was 97.56%. In the sagittal plane, mean accuracy was 97.59%, sensitivity was 97.50%, and specificity was 97.67%. In this respect, a statistically significant diagnosis of myelopathy was made in both axial and sagittal planes. However, it is seen that the rates of myelopathy detection in our study are higher than those of other studies in the literature.

The present study has some limitations. The retrospective nature of the study and small sample size can be considered as limitations. More participants could be included in the study and more accurate information could be obtained in determining the accuracy rate. On the other hand, there are also strengths of the study. The cervical MRIs of the participants with myelopathy symptoms taken in a single center and with the same MRI device were evaluated by two physicians experienced in spinal diseases. In this way, standardization of the images was tried to be kept at the maximum level. In addition, since there could be a difference between MRI sequences taken even in the same participant, normal and the area of myelomalacia images were compared on the same sections.

In the last half century, ML and artificial neural networks (ANN) are frequently preferred in solving nonlinear problems. The DL method, which is a multi-layered ANN, is widely used in the field of health, with the increasing computational power of computers and the easier digital acquisition of medical images. Human-induced errors can be reduced with high accuracy and fast prediagnosis systems to be revealed. In subsequent studies based on the present study, a pre-diagnosis system can be established by automatic detection of the region with anomaly, staging and monitoring of the myelopathy status can be achieved.

## Conclusion

Machine learning technologies are used in many health applications, especially in medical image analysis. Deep learning, a sub-branch of ML, has shown a great improvement especially in spine surgery. In the present study, we aimed to reveal the effectiveness of DL in the diagnosis of CM. As a result of our study, we observed that DL technology works with a higher accuracy than other studies in the literature for the diagnosis of CM. However, in subsequent studies, a prediagnosis system can be established by automatic detection of the region with anomaly, staging, and monitoring of the myelopathy status can be provided.
