# Supplementary material for: Convolutional Neural Networks in the Diagnosis of Cervical Myelopathy
Source: Rev Bras Ortop (Sao Paulo). 2024 Dec 7;59(5):e689–95. doi: 10.1055/s-0044-1779317 (PMC11624937; doi:10.1055/s-0044-1779317)
Supplement: Related texts

# Research article: Redes neurais convolucionais no diagnóstico de mielopatia cervical

**Keywords:** aprendizado profundo, imagem por ressonância magnética, mielopatia, redes neurais artificiais, vértebras cervicais

## Abstract

**Objetivo** As tecnologias de inteligência artificial são cada vez mais utilizadas em cirurgias de coluna como ferramentas diagnósticas. O objetivo do presente estudo foi avaliar a eficácia das redes neurais convolucionais no diagnóstico da mielopatia cervical (MC) em comparação à ressonância magnética (RM) cervical convencional.

**Métodos** O presente estudo foi transversal, descritivo e analítico. Cento e vinte e cinco participantes com diagnóstico clínico e radiológico de MC foram incluídos no estudo. Foram utilizadas imagens de RM sagital e axial em sequência ponderada em T2 da coluna cervical. Todas as imagens foram obtidas em 8 bits/pixel em duas categorias diferentes (MC e normal), tanto em vistas axiais quanto sagitais.

**Resultados** A validação transversal tripla evitou o sobreajuste ( *overfitting* ) durante o processo de treinamento. Duzentas e quarenta e duas imagens foram utilizadas para treinamento e teste do modelo criado para vistas axiais, que apresentou 97,44% de sensibilidade e 97,56% de especificidade. Duzentas e quarenta e nove imagens foram utilizadas para treinamento e teste do modelo criado para vistas sagitais, que apresentou 97,50% de sensibilidade e 97,67% de especificidade. Após o treinamento, a acurácia média foi de 96,7% (±1,53) para a vista axial e de 97,19% (±1,2) para a vista sagital.

**Conclusão** O *deep learning* (DL) apresentou grande melhora, especialmente na cirurgia de coluna. Observamos que a tecnologia de DL trabalha com maior acurácia do que em outros estudos na literatura para diagnóstico de MC.

## Introdução

A mielopatia cervical (MC) é uma doença degenerativa comum da coluna cervical causada pela compressão da medula espinhal. 1 De modo geral, a diminuição do volume do canal espinhal se deve à degeneração do disco, osteófitos ou ossificação do ligamento longitudinal e é acompanhada por alterações isquêmicas. Os achados clínicos à MC variam conforme a gravidade e a localização da compressão medular. 2 Os pacientes apresentam dormência e dor nas extremidades e sintomas neurológicos, como perda de coordenação e equilíbrio. Os achados em modalidades clínicas e radiológicas devem ser avaliados em conjunto para diagnóstico e determinação das opções terapêuticas. 3

As técnicas de imagem para diagnóstico da MC são radiografia simples, ressonância magnética (RM) e tomografia computadorizada (TC). Na MC, a RM tem mais valor para avaliação da medula espinhal, do disco e de outros tecidos moles em comparação com outros métodos de imagem. 4 5 Os avanços tecnológicos na RM, como a espectroscopia por RM e a técnica por tensor de difusão, melhoraram muito a resolução e a qualidade da imagem. Além disso, novos métodos de imagem estão sendo desenvolvidos para diagnóstico da MC. 6

As redes neurais convolucionais (CNNs, do inglês *convolutional neural networks* ), uma técnica de aprendizado de máquina (ML, do inglês *machine learning* ), são cada vez mais utilizadas em vários campos industriais e de pesquisa. 7 O *deep learning* (DL) é uma rede neural multicamadas com extração automática de recursos. Estende as redes neurais tradicionais ao adicionar mais camadas ocultas à arquitetura da rede, entre as camadas de entrada e saída, para modelamento de relacionamentos mais complexos e não lineares.

Nos últimos anos, as tecnologias de ML também passaram a ser empregadas em muitas aplicações de saúde, como análise de imagens médicas, sinais biológicos e registros de saúde, previsão de eventos médicos, detecção e diagnóstico auxiliados por computador e escolha de tratamentos. 8

A tecnologia de ML é cada vez mais usada em cirurgias de coluna e em muitas áreas médicas. Porém, percebe-se que a maioria dos estudos sobre este assunto na literatura não foram realizados na coluna cervical. 9 No presente estudo, objetivamos demonstrar a eficácia da CNN no diagnóstico de MC em comparação com a RM cervical convencional.

## Materiais e Métodos

## Participantes e amostras de RM

Participantes com 18 a 75 anos de idade e diagnóstico de MC foram incluídos no estudo. Todas as imagens foram obtidas com equipamento de 3.0 Tesla Siemens (Siemens, Erlangen, Alemanha) e colar cervical padrão. As imagens radiológicas da coluna cervical foram avaliadas por três investigadores com experiência mínima de 5 anos em cirurgia da coluna vertebral. Foram utilizadas imagens de RM axial e sagital da coluna cervical. As imagens em formato de Comunicação de Imagens Digitais em Medicina (DICOM, do inglês Digital Imaging and Communications in Medicine) (8 bits/pixel) foram convertidas para um dos formatos *raster* (png). A partir daí, foi feita a seleção das “regiões de interesse”, que foram rotuladas como MC e normais, tanto nas vistas axiais quanto sagitais ( Fig. 1 ).

**Fig. 1:** Região marcada de interesse nas imagens axiais e sagitais de ressonância magnética (acima) e nas amostras de imagens de entrada (abaixo).

Os critérios de inclusão do estudo foram participantes com sintomas clássicos de MC, como fraqueza e inépcia em extremidades superiores, hiperreflexia e/ou dificuldades de marcha, achados radiológicos de compressão espinhal e escores da escala modificada da Japanese Orthopedic Association (mJOA) > 18. Participantes com histórico de cirurgia da coluna cervical e portadores de doença sistêmica (doença reumatológica ou neurológica) foram excluídos. O estudo foi aprovado pelo comitê de ética local (KAEK/2020.07.129) e registrado em Clinicaltrials.gov (NCT04796987).

## Ambiente de cálculo

Um computador com 8 GB de memória e processador Intel i7 realizou os cálculos e o treinamento do modelo de DL. Os modelos de DL foram desenvolvidos com a linguagem de programação Python. A avaliação do desempenho do modelo foi baseada em acurácias transversais validadas médias e medianas, sensibilidade, especificidade e valor preditivo positivo e negativo.

## Projeto de rede neural

Desenvolvimentos significativos nas técnicas de ML e de processamento de imagens permitem a avaliação mais precisa das imagens médicas com métodos de DL. 7 O termo “aprendizado profundo” é empregado há muitos anos na classificação de imagens médicas. A CNN, um dos métodos populares de DL, classificou as imagens deste estudo. Uma arquitetura CNN simples consiste em camada convolucional (Conv), camada de *pooling* (por exemplo, *pooling* máximo), camada não linear (por exemplo, ReLU, TanH) e uma função de perda (por exemplo, sigmoide, Softmax) na última camada totalmente conectada (FC, do inglês *fully connected* ). A saída pode ser de uma única classe, mas também pode ser múltipla. 10 A Fig. 2 mostra a arquitetura de CNN usada no presente estudo.

**Fig. 2:** Arquitetura de redes neurais convolucionais (CNNs) usada neste estudo.

## Resultados

Cento e vinte e cinco participantes foram incluídos no estudo, sendo 58 do sexo feminino e 67 do sexo masculino, com idade média de 56,32 ± 6,77 anos e diagnóstico de MC. O escore mJOA médio dos participantes foi de 12,01 ± 2,46. A Tabela 1 mostra as características demográficas dos participantes. As camadas normais e com mielomalácia das RMs dos participantes foram analisadas.

**Tabela 1:** Dados demográficos e características clínicas dos participantes

Variável	Participantes ( *n* = 125)
Idade (em anos) (média ± desvio padrão)	56,32 ± 6,77
Sexo (feminino/masculino) n (%)	58 (46,4%)/67 (53,6%)
Altura (cm) (média ± desvio padrão)	164,5 ± 8,52
Peso (kg) (média ± desvio padrão)	74,96 ± 8,48
Pontuação na escala mJOA	12,01 ± 2,46

Abreviações: cm, centímetros; kg, quilograma; mJOA, escala modificada da Japanese Orthopedic Association.

## Informações de treinamento

Imagens obtidas em vista axial e sagital foram utilizadas para treinamento do projeto de rede criado. Assim, foram realizados dois treinos diferentes de DL para ambas as vistas. Sessenta e sete por cento das imagens foram utilizadas para treinamento e 33% para validação e teste. A validação transversal tripla testou a consistência dos resultados.

Os modelos obtidos de ambas as vistas têm 300 iterações de treinamento ( *epochs* ). A validação transversal tripla evitou o sobreajuste ( *overfitting* ) durante o processo de treinamento. Após o treinamento, a acurácia média foi 96,7% (±1,53) para a vista axial e 97,19% (±1,2) para a vista sagital. A Fig. 3 mostra os gráficos do processo de treinamento de DL.

**Fig. 3:** Informações de treinamento das vistas sagital e axial da ressonância magnética.

## Avaliação das métricas de treinamento

## Vista axial

No total, 242 imagens de amostra foram utilizadas para treinamento e teste do modelo criado para vistas axiais. Destas imagens, 120 foram rotuladas como MC e 122 como normais. Ao todo, 162 dessas imagens foram utilizadas para o treinamento do modelo de DL e 80 para avaliação do treinamento. A Tabela 2 mostra os valores de verdadeiro-positivo, verdadeiro-negativo, falso-positivo e falso-negativo obtidos na avaliação do treinamento. A Fig. 4 mostra a matriz de confusão formada por esses de acurácia. Depois da avaliação, a acurácia geral do modelo foi 97,50%.

**Fig. 4:** Matriz de confusão da vista axial (à esquerda) e da vista sagital (à direita).

**Tabela 2:** Detalhes das imagens sagitais e axiais usadas para avaliação

População total		Humanos (condição verdadeira)	
		*Condição positiva*	*Condição negativa*
Modelo desenvolvido (condição prevista) para imagens sagitais	*Condição prevista positiva*	Verdadeiro-positivo = 39	Falso-positivo = 1 (erro de tipo I)
	*Condição prevista negativa*	Falso-negativo = 1 (erro de tipo II)	Verdadeiro-negativo = 42
Modelo desenvolvido (condição prevista) para imagens axiais	*Condição prevista positiva*	Verdadeiro-positivo = 38	Falso-positivo = 1 (erro de tipo I)
	*Condição prevista negativa*	Falso-negativo = 1 (erro de tipo II)	Verdadeiro-negativo = 40

A taxa de verdadeiro-positivos (TVP, *recall* , sensibilidade) foi 97,44%, a taxa de verdadeiro-negativos (TVN, especificidade) foi 97,56%, a taxa de falso-negativos (TFN, taxa de erros) foi 2,56% e o valor preditivo positivo (VPP, precisão) foi 97,44%. Além disso, a pontuação F1 foi 0,97, o coeficiente de correlação de Matthew (CCM) foi 0,95 e o coeficiente *kappa* de correlação de Cohen foi 0,95.

Após a avaliação, a curva de característica de operação do receptor (ROC, do inglês *receiver operating characteristic* ) foi traçada e a área sob a curva (AUC, do inglês *area under the curve* ) ROC 11 foi calculada ( Fig. 5 ).

**Fig. 5:** Curva de característica de operação do receptor (ROC) e área sob a curva (AUC) da vista axial (à esquerda) e da vista sagital (à direita).

## Vista sagital

Duzentas e quarenta e nove imagens de RMs dos participantes foram selecionadas para treinamento e teste do modelo criado para vistas sagitais. Destas imagens, 124 foram rotuladas como MC e 125 como normais. Cento e sessenta e seis dessas imagens foram utilizadas para o treinamento do modelo de DL e 83 para avaliação do treinamento. A Tabela 2 mostra os valores de verdadeiro-positivos, verdadeiro-negativos, falso-positivos e falso-negativos após a avaliação do treinamento. A Fig. 4 mostra a matriz de confusão formada por esses de acurácia. Depois da avaliação, a acurácia geral do modelo foi calculada em 97,59%.

A TVP ( *recall* , sensibilidade) foi 97,50%, a TVN (especificidade) foi 97,67%, a TFN (taxa de erros) foi 2,50% e o VPP (acurácia) foi 97,50%. Além disso, a pontuação F1 foi 0,98, o CCM foi 0,95 e o coeficiente *kappa* de correlação de Cohen foi 0,95.

Após a avaliação, a curva ROC foi traçada e a AUC foi calculada ( Fig. 5 ).

## Discussão

As tecnologias de ML começaram a ser utilizadas em muitas áreas devido ao seu desenvolvimento nos últimos anos. 12 O DL, um sub-ramo do ML, tem melhorado muito, em especial na área médica. Paralelamente a esse desenvolvimento, o uso de DL em cirurgias de coluna aumentou de forma significativa nos últimos anos e tem aprimorado a determinação de problemas de coluna e a avaliação de opções terapêuticas. 9 Hoje, o ML é empregado com maior frequência na avaliação de diagnósticos por imagem, como a localização das vértebras e dos discos e a determinação do formato da coluna, a partir de informações obtidas em métodos radiológicos, como a TC e a RM. 13 O progresso nos estudos de ML está aumentando gradativamente sua utilização em protocolos de tratamento e previsão de possíveis resultados. 14

O diagnóstico de MC é baseado nos achados obtidos no exame clínico e nas imagens radiológicas. A RM é o método diagnóstico mais eficaz para avaliação radiológica de pacientes com sintomas clínicos mielopáticos. 15 A anatomia da medula espinhal e das estruturas do canal medular são mais bem avaliadas nas vistas sagitais e axiais. 16 17 A MC manifesta-se caracteristicamente como atrofia medular focal hipointensa na sequência ponderada em T1 e hiperintensa na sequência ponderada em T2. No entanto, há relatos que a sequência T2 detecta melhor alterações intramedulares e é mais valiosa em termos de prognóstico. 15 Também foi relatado que de 58 a 85% dos pacientes com sintomas clínicos de MC apresentam sinal hiperintenso na sequência T2. 18 O diagnóstico da MC baseia-se principalmente em análises subjetivas dos achados à RM da medula espinhal e, portanto, é limitado pelo treinamento e pela experiência do avaliador. 19 Portanto, no presente estudo, tentamos revelar a eficácia do DL no diagnóstico da mielopatia para eliminar este aspecto subjetivo.

No presente estudo, avaliamos a sequência ponderada em T2 da RM cervical dos participantes com mielomalácia. Obtivemos cortes sagitais e axiais de RM em sequência T2 dos participantes, marcamos as áreas normais e de mielomalácia na região intramedular e treinamos o equipamento. A RM cervical dos participantes foi realizada em um único centro e com o mesmo equipamento. No entanto, como as imagens de RM podem diferir de paciente para paciente, as imagens normais e do campo da mielomalácia foram obtidas no mesmo corte para padronizá-las.

Um estudo piloto analisou a eficácia do ML no diagnóstico de MC em 28 pacientes. O estudo avaliou RMs cervicais de participantes com sintomas de mielopatia e obteve acurácia mediana de 90%. A sensibilidade média foi 90% e a especificidade, 85%. 20 Da mesma forma, outro estudo que investigou a eficácia do ML na MC utilizou imagens obtidas com tensor de difusão, obtendo acurácia média de 89,7%, sensibilidade de 85% e especificidade de 92,4%. 21 Em outro estudo, a maior acurácia média encontrada foi 77%, a sensibilidade média foi 78% e a especificidade média foi 80%. 22 Em nosso estudo, utilizamos RMs cervicais convencionais, o padrão diagnóstico na rotina diária. Como a determinação da anatomia da medula espinhal é melhor nos planos axial e sagital, avaliamos esses dois planos de maneira separada. Ao final do estudo, a acurácia mediana no plano axial foi de 97,50%. A sensibilidade média foi 97,44% e a especificidade foi 97,56%. No plano sagital, a acurácia média foi 97,59%, a sensibilidade foi 97,50% e a especificidade foi 97,67%. O diagnóstico de mielopatia foi realizado de forma estatisticamente significativa nos planos axial e sagital. Porém, verifica-se que as taxas de detecção de mielopatia em nosso estudo são superiores às de outros relatos da literatura.

O presente estudo tem algumas limitações, como sua natureza retrospectiva e o pequeno tamanho da amostra. O número de participantes poderia ser maior, assim como a precisão das informações para determinação da taxa de acurácia. Por outro lado, o estudo também apresenta pontos fortes. As RMs cervicais de participantes com sintomas de mielopatia, realizadas em um único centro e com o mesmo equipamento, foram avaliadas por dois médicos experientes em doenças da coluna vertebral. Desta forma, procurou-se manter ao máximo a padronização das imagens. Além disso, como poderia haver uma diferença entre as sequências de RM obtidas no mesmo participante, as imagens normais e da área de mielomalácia foram comparadas nos mesmos cortes.

No último meio século, o ML e as redes neurais artificiais (ANN, do inglês *artificial neural networks* ) são preferidos na resolução de problemas não lineares. O método de DL, que é uma ANN multicamadas, é amplamente utilizado na área da saúde, aumentando o poder computacional e a facilidade de aquisição digital de imagens médicas. Erros induzidos por humanos podem ser reduzidos com alta precisão, gerando sistemas rápidos de pré-diagnóstico. Estudos subsequentes podem vir a estabelecer um sistema de pré-diagnóstico por meio da detecção automática da região com anomalia, permitindo o estadiamento e o monitoramento da mielopatia.

## Conclusão

As tecnologias de ML são usadas em muitas aplicações de saúde, especialmente em análise de imagens médicas. O DL, um sub-ramo do ML, tem apresentado grande melhora principalmente na cirurgia de coluna. Neste estudo, objetivamos revelar a eficácia do DL no diagnóstico de MC. Como resultado do nosso estudo, constatamos que a tecnologia de DL funciona com maior precisão do que outros estudos da literatura para o diagnóstico de MC. No entanto, em estudos subsequentes, um sistema de pré-diagnóstico pode ser estabelecido pela detecção automática da região com anomalia, podendo levar ao estadiamento e monitoramento da mielopatia.
